# Infection risk by oral contamination does not induce immune priming in the mealworm beetle (*Tenebrio molitor*) but triggers behavioral and physiological responses

**DOI:** 10.3389/fimmu.2024.1354046

**Published:** 2024-02-08

**Authors:** Alexandre Goerlinger, Charlène Develay, Aude Balourdet, Thierry Rigaud, Yannick Moret

**Affiliations:** CNRS UMR 6282 Biogéosciences, Université de Bourgogne, Dijon, France

**Keywords:** *Tenebrio molitor*, evolution of immune priming, oral infection, behavioral defense, gut immunity, entomopathogen, bacteria

## Abstract

In invertebrates, immune priming is the ability of individuals to enhance their immune response based on prior immunological experiences. This adaptive-like immunity likely evolved due to the risk of repeated infections by parasites in the host’s natural habitat. The expression of immune priming varies across host and pathogen species, as well as infection routes (oral or wounds), reflecting finely tuned evolutionary adjustments. Evidence from the mealworm beetle (*Tenebrio molitor*) suggests that Gram-positive bacterial pathogens play a significant role in immune priming after systemic infection. Despite the likelihood of oral infections by natural bacterial pathogens in *T. molitor*, it remains debated whether ingestion of contaminated food leads to systemic infection, and whether oral immune priming is possible is currently unknown. We first attempted to induce immune priming in both *T. molitor* larvae and adults by exposing them to food contaminated with living or dead Gram-positive and Gram-negative bacterial pathogens. We found that oral ingestion of living bacteria did not kill them, but septic wounds caused rapid mortality. Intriguingly, the consumption of either dead or living bacteria did not protect against reinfection, contrasting with injury-induced priming. We further examined the effects of infecting food with various living bacterial pathogens on variables such as food consumption, mass gain, and feces production in larvae. We found that larvae exposed to Gram-positive bacteria in their food ingested less food, gained less mass and/or produced more feces than larvae exposed to contaminated food with Gram-negative bacteria or control food. This suggests that oral contamination with Gram-positive bacteria induced both behavioral responses and peristalsis defense mechanisms, even though no immune priming was observed here. Considering that the oral route of infection neither caused the death of the insects nor induced priming, we propose that immune priming in *T. molitor* may have primarily evolved as a response to the infection risk associated with wounds rather than oral ingestion.

## 1 Introduction

Immune priming is a phenomenon observed in invertebrates wherein individuals acquire increased resistance to infections following a first non-lethal contact with a pathogen or an antigen. The initial evidence of immune priming is credited to Huang and Song, who demonstrated that a species of shrimp (*Penaeus monodon*) developed an enhanced resistance to a virus after being injected with yeast glucans (1). The authors paved the way for numerous studies that provided evidence of immune priming in other invertebrate species. Both specific and non-specific immunity were evidenced after homologous (e.g.,: 2, 3) or heterologous challenges (e.g.,: 1, 4). Further studies also showed that immune priming is not ubiquitous across all invertebrate-pathogen interactions (e.g.,: 5). In the majority of these studies, immune priming was induced by septic wounding or direct injection of pathogens into the hemocoel (6, 7). However, this method may not always be ecologically relevant, as many parasites and pathogens infect their host through the oral route (2, 8). Moreover, the dynamics of infection are expected to widely vary depending on the infection route: infection through a wounded cuticle can rapidly spread throughout the whole body and lead to death by septicemia, whereas pathogens infecting their host by the oral route first need to pass through the gut barrier to reach the hemocoel or can be eliminated efficiently by the host (9). Therefore, some studies found that eating contaminated food could trigger immune priming (2, 10, 11), but others found that the efficiency of priming may depend on the route of infection, leading to different evolutionary pathway for immune priming (12). Testing different routes of infection could yield differences in outcomes that are necessary to fully understand the ecological and evolutionary implications of immune priming.

Among the invertebrate species, the mealworm beetle (*Tenebrio molitor*) has rapidly become a model for studying immune priming (see (7, 13, 14) for reviews). After a primary immune challenge with inactivated Gram(-) or Gram(+) bacteria, the immune response remains activated long enough to protect the insect against a subsequent infection with the same or a different microbial pathogen than the one used to trigger the primary response (4, 15). However, challenges with Gram(+) bacterial pathogens stimulated a more protective priming response in terms of efficiency and duration against subsequent infections (15), suggesting that this range of bacterial pathogens may have been a relatively significant selective force for the evolution of immune priming in this insect species. However, all these previous studies used injections as a proof of concept to mimic primary contacts with bacterial pathogens, without considering potential variations in infection routes. This species generally lives in enclosed environments at high densities, with densities even increasing in companies that now produce this insect as a new source of proteins (16). Cannibalism is frequently observed in *T. molitor* in both larvae and adults (17, 18), as well as numerous bites and fights (19, 20). Due to these traits, injuries are frequently observed, leading to a high risk of repeated infections by this route and the long-term maintenance of infections in populations. However, due to cannibalism and the fact that *T. molitor* feeds on its living environment (bran, wheat flour, etc.), the ingestion of corpses or food contaminated by cadavers is probably common. The oral route can therefore logically be considered as another relevant natural route of transmission of pathogens. To our knowledge, only a few recent studies have explored infection or immune priming by the oral route in *T. molitor*. Zanchi et al. (21) successfully infected *T. molitor* by feeding them with spores of *Bacillus thuringiensis*, while Dupriez et al. (16) found that cultures of *Serratia marcescens* persist several days in the bran medium and increase mortality in *T. molitor* living in this contaminated food. These studies suggest that ecological conditions for oral pathogen infection and transmission exist for *T. molitor*. In addition, González-Acosta et al. (22) claimed to have successfully primed larvae by feeding them with killed bacterial pathogens. However, in this study, priming was referred to as a better survival at a secondary infection occurring only 24 hours after the feeding period; therefore, the slightly better resistance of larvae could be attributed to a short-term elevation of immunity rather than a phenomenon of immune priming. Indeed, a successful immune priming implies that individuals can better resist a second infection even when it occurs several days after the immunization (3, 15).

It is noteworthy that the insect gut is capable of rapidly mounting defense reactions upon detection of ingested bacteria, such as strong intestinal contractions (23) and enterocyte purge (24), as observed in *Drosophila melanogaster*. Beforehand, insects can also detect entomopathogens in their food, exhibiting avoidance behavior towards contaminated food. This behavior was observed, for example, in *Bombus terrestris* exposed to the trypanosome *Crithidia bombi* (25), and even in *T. molitor* larvae exposed to the fungi *Fusarium avenaceum* and *Beauveria bassiana* (26). Therefore, it is pertinent to consider not only the immune component of resistance but also physiological or behavioral reactions to exposure to contaminated food, as they could be of great interest.

It is known that immune defenses vary with developmental stage in several insect systems (27). One may predict larvae to exhibit stronger immune reactions than adults, considering that sexually mature individuals must allocate their energy expenditure between maintenance and reproduction, leading to a decreasing in the efficiency of the immune system with age, as observed, for instance, in *Anopheles gambiae* (28). Consistently, in *T. molitor*, it seems that the phenoloxidase activity and the number of hemocytes (two major compounds of invertebrate immunity) were higher in larvae than in adults (29). Therefore, comparing the resistance of larvae and adults to a second infection after priming by the oral route should provide valuable insights.

In this study, we aimed to induce immune priming by the oral route in *T. molitor* larvae and adults by feeding them food contaminated with two bacteria species. Subsequently, we compared the individuals’ resistance to a second infection involving the same pathogen that was used for feeding. In addition, we controlled the amount of contaminated food ingested by larvae by analyzing their feces after the feeding period, using four bacteria species. We hypothesized that Gram(+) bacteria should trigger the strongest reactions in comparison to Gram(-) bacteria, as already observed after septic wounding in *T. molitor* (15). Our results revealed that feeding with the Gram(+) bacteria *Bacillus cereus* and *Staphylococcus aureus* triggered increased defecation in larvae when compared to the Gram(-) bacteria *Serratia marcescens* and *Escherichia coli*, and that larvae fed with *B. cereus* decreased their feeding rate. However, consuming contaminated food only protected the adults, and only briefly, when the second infection occurred right after the feeding period. We did not observe any long-term resistance to a second infection, which suggests that oral contamination may therefore not be the major route through which immune priming evolved in *T. molitor.*

## 2 Material and methods

### 2.1 Insects

*Tenebrio molitor* insects from an outbred stock were reared in permanent obscurity at 24 ± 1°C and 70 ± 10% relative humidity. They were fed with wheat bran supplemented with apples. To ensure a constant supply of larvae of known age, we routinely isolated 50 adult beetles of each sex in separate tanks and allowed them to reproduce for five days. For the experiments, larvae aged 12 weeks (determined from the beginning of each breeding period) were sampled, while adults were collected 10 days after emergence. Ethical review and approval were not required for the study on these animals in accordance with the local legislation and institutional requirements.

### 2.2 Bacterial cultures

The following tetracycline-resistant bacterial strains were obtained from the collection of the Pasteur Institute: *Bacillus cereus* (CIP 69.12, Gram(+), entomopathogen), *Staphylococcus aureus* (CIP 52.149, Gram(+), generalist), *Serratia marcescens* (CIP 103235T, Gram(-), entomopathogen), and *Escherichia coli* (CIP 103470, Gram(-), generalist) (respectively Bc, Sa, Sm, and Ec). All bacteria were stored at -80°C in 500 µL aliquots of Broth medium (10 g.L^-1^ bactotryptone, 5 g.L^-1^ yeast extract, 10 g.L^-1^ NaCl, pH = 7.5) mixed with glycerol (86%) at a 3:2 ratio. Prior to use, bacterial samples were thawed on ice and cultured overnight in Broth medium with added tetracycline (5 µg.mL^-1^ T3383, Sigma-Aldrich) in an incubator at 28°C and 200 rpm agitation. Subsequently, each bacterial suspension was evenly spread with a plastic spreader on a Petri dish containing a mixture of 1% agar, Broth medium, and tetracycline (5 µg.mL^-1^). Petri dishes were then incubated overnight at 28°C. Finally, new bacterial suspensions were made by harvesting 1 colony-forming unit (CFU) from each Petri dish, and these were cultured in 40 mL of Broth medium without tetracycline under the same conditions as described above. This protocol ensured that the bacterial suspensions remained uncontaminated by other bacteria.

### 2.3 First experiment: oral priming with living or inactivated bacteria

#### 2.3.1 Feeding

For this experiment, we collected 540 larvae and 700 adults (350 males and 350 females) from our stock. Individuals were weighed to the nearest 1 mg (thereafter referred to as initial mass) using an OHAUS balance (discovery series, DU114C) and isolated in compartmented boxes (boxes with 10 compartments; each compartment: L × 1 × H = 4.8 × 3.2 × 2.2 cm) for two days without food. They were then fed as follows.

To replicate the experimental conditions of González-Acosta et al. (22) wherein a short-term priming effect was observed in *T. molitor* larvae, we provided the animals with shredded carrots soaked in a bacterial suspension. Specifically, we used *B. cereus* and *S. marcescens*, as preliminary experiments indicated that these two strains exhibited the highest virulence for the larvae, thereby potentially maximizing the likelihood of a priming response. To prepare the diet contaminated with living bacteria, bacterial suspensions were prepared as described earlier, washed twice with phosphate-buffered saline (PBS 10 mM, pH = 7.4), and adjusted to 10^9^ cells.mL^-1^ by using a hemocytometer under a microscope (magnification: x400). Organic carrots were minced with an electric mincer and added to the bacterial suspensions (2 g of carrots per mL of suspension). For the diet contaminated with inactivated bacteria, bacterial suspensions were adjusted to 10^6^ cells.mL^-1^ and microwaved until boiling before the same amount of minced carrots was added. A different concentration was used for inactivated bacteria to match the protocol of González-Acosta et al. (22), but inactivated bacteria at 10^9^ cells.mL^-1^ were also used in adults. The control diet was prepared by mixing carrots with sterile Broth medium.

The diet was provided *ad libitum* for 24 hours in compartmented boxes without additional food. The carrots were then removed, and wheat bran was provided *ad libitum*.

#### 2.3.2 Challenge with living bacteria through septic wounding

New bacterial suspensions were prepared as previously described (without washing with PBS), and they were centrifuged at 4000 rpm for 10 minutes to obtain bacterial pellets. Individuals were anesthetized on ice for 10 minutes before being wounded with a small needle (^©^Pic Solution) dipped in a bacterial pellet. The individuals were pricked with the same bacterial strain they ingested during the feeding period. For the two bacteria strains tested, 80 larvae and 80 adults (40 males and 40 females) fed with either a control diet, a diet contaminated with living bacteria, or a diet contaminated with inactivated bacteria received septic wounds. Additionally, 60 larvae and 60 adults fed a control diet received sterile wounds as procedural controls or were left unwounded (**Supplementary Table S1**). Individuals were not all wounded at the same time. For each group, one half (i.e., 40 individuals) was pricked immediately at the end of the feeding period (referred to as 24 hours post-feeding), while the other half was pricked two weeks later (referred to as 15 days post-feeding, **Supplementary Table S1**). After wounding, individuals were returned to compartmented boxes without food for 24 hours to prevent healing problems that may arise when wheat bran sticks to the open wound. Subsequently, individuals were fed with wheat bran *ad libitum*, and survival was monitored daily for two weeks post-wounding.

### 2.4 Second experiment: monitoring the ingestion of living bacteria by larvae and its effects

#### 2.4.1 Control of the amount of ingested food

For this second experiment, we collected 360 larvae from our stock. They were weighed (hereafter referred to as initial mass) and isolated in compartmented boxes for two days without food.

To prepare the contaminated diet, bacterial suspensions were prepared as described earlier, washed twice with PBS, resuspended in Broth medium, and adjusted to 2x10^9^ cells.mL^-1^. Agar at 2% in Broth medium was autoclaved at 105°C for 18 minutes. Subsequently, the bacterial suspensions (Bc, Sa, Sm, or Ec) were added to the Broth-agar mix at a 1:1 ratio, resulting in a concentration of 10^9^ cells.mL^-1^ and 1% agar. These preparations were conducted in a water bath at 45°C. This temperature was selected to prevent the Broth-agar mix from solidifying too quickly while remaining tolerable for living bacteria. Twenty microliters of each preparation were pipetted into 1.5 mL centrifuge tubes with the cap and side previously pierced with a small drill (Dremel, 1 mm) to allow larvae to breathe while being inside. Before putting larvae into the tubes, the latter were weighed at the nearest hundredth of a milligram (Sartorius Lab Instruments GmbH & Co. KG). In total, 160 tubes containing contaminated diet (40 tubes per bacteria species) were prepared. Additionally, 200 control tubes were prepared by replacing the bacterial suspension with sterile Broth medium (**Supplementary Table S2**).

Larvae were introduced into the tubes and allowed to feed for 24 hours. They were then transferred into new tubes prepared as described above. Tubes were weighed again after feeding to calculate the amount of food consumed by each larva. This protocol was repeated with three sets of tubes. This experiment was not conducted with adults due to technical constraints, primarily because feeding in the tubes could not be precisely controlled.

#### 2.4.2 Bacteria in the feces

At the end of the third day of feeding, we collected feces from all larvae by transferring them into empty, pre-weighed pierced tubes. After 24 hours, larvae were removed from the tubes and weighed again so we could calculate their mass gain or loss over the feeding period. These values were normalized by dividing them by the individual’s initial mass. Among the 360 tubes, 100 were randomly selected (20 per treatment) to determine the mass of feces produced by the corresponding individuals during the 24-hour period. Specifically, 20 tubes were selected from larvae fed on the control diet, and additional 20 tubes were sampled from larvae fed on each diet contaminated with either Bc, Sa, Sm, or Ec. For each individual, fecal sample was homogenized in 1 mL of PBS, and 300 µL of this mixture was spread on 3 Petri dishes (100 µL per Petri dish) containing a mix of 1% agar and tetracycline (5 µg.mL^-1^). Bacteria were cultured overnight at 28°C, and CFUs were subsequently counted using an automatic colony counter (Interscience Scan 500).

#### 2.4.3 Challenge with living bacteria through septic wounding

After feces collection, larvae were kept in compartmented boxes with wheat bran under the same conditions as described above. Larval survival was monitored daily for two weeks. Subsequently, larvae, which were 15 weeks old at this point (that is 2 weeks after the feeding treatment), were subjected to a challenge through septic wounding, following the procedure described earlier (i.e., wounding with a syringe contaminated by living bacteria with the same strain they ingested during the feeding period). Additionally, 20 control larvae were punctured with a sterile syringe, and another 20 control larvae were left unwounded to account for the potential impact of wounding alone (**Supplementary Table S2**).

After the wounding process, larvae were placed back in compartmented boxes without food for 24 hours and were then fed with wheat bran *ad libitum*. Larval survival was monitored daily for two weeks.

### 2.5 Statistics

All statistics were done with R studio (V. 2023.06.0).

Individual survival was analyzed with Cox models (using the ‘coxme’ package). In all our models, the bacteria species (*B. cereus*/*S. marcescens*) and the time of septic wounding (24 hours and 15 days post-feeding) were fixed factors, as well as the type of food. Depending on the data analyzed, the type of food varied. This included control food, contaminated food with inactivated bacteria at 10^6^ cells.mL^-1^, and contaminated food with living bacteria at 10^9^ cells.mL^-1^ when comparing larvae and adult together. For the analysis involving only adults, the type of food considered as a fixed factor included control food, contaminated food with inactivated bacteria at 10^6^ cells.mL^-1^, contaminated food with inactivated bacteria at 10^9^ cells.mL^-1^, and contaminated food with living bacteria at 10^9^ cells.mL^-1^. Additionally, the development stage (larva and adult, when comparing larvae and adults) or sex (male and female, when considering only the adults) was included as a fixed factor. The initial body mass (or initial mass nested into the development stage in the global model) was considered a continuous covariate to control for a putative effect of animal quality, and the box in which the insects were maintained was considered a random factor. In all of our analyses, all possible two-way interaction terms between factors were kept in the final model, while three-way interaction terms were retained only if they were found to be significant. Individuals that died before the wounding treatments were not considered, and individuals that were still alive at the end of the monitoring periods were censored.

Both the quantity of food ingested and feces produced by the larvae were analyzed using linear models, as homoscedasticity conditions were met for both analyses. For food, a linear mixed-effects model was used because the boxes in which insects were maintained were included as a random factor. For feces analyses, boxes were not considered since only a random subsample of larvae was used, and some boxes were not represented. For both analyses, the type of food (i.e., the bacteria species included in the food plus the control without bacteria) and the initial body mass of individuals were considered as fixed factors.

The number of CFUs present within the feces was analyzed using a generalized linear model with a quasi-Poisson distribution and a log link function. The bacterial content of the food (i.e., the species of bacteria included within the food) was treated as a fixed factor, while the quantity of food ingested, feces mass, and individual initial body mass were continuous covariates.

## 3 Results

Almost no mortality was observed during the period from feeding to wounding in both of our experiments, among larvae (global survival rate: 99.9%) or adults (global survival rate: 98.4%).

### 3.1 First experiment: oral priming and subsequent infections

Among the control individuals (fed with uncontaminated food), mortality due to the sterile wounding was stage-dependent. This treatment had no effect on larval mortality: survival was 100% at the end of the two-week monitoring in all groups. However, in adults, survival was significantly lower 15 days after wounding with the sterile needle compare to other groups (**Supplementary Figure S1**; **Supplementary Table S3**).

**Figure 1:**
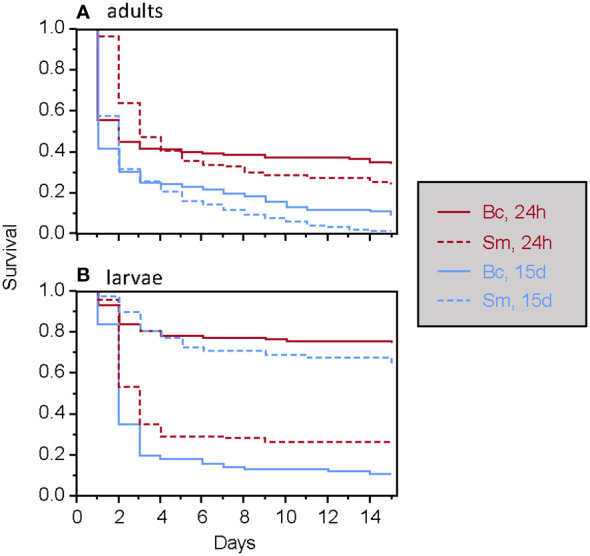
Survival of *T. molitor* after a secondary septic wound occurring either 24 hours or 15 days after the feeding treatment among adults **(A)** and larvae **(B)**, according to the bacteria species. Bc, *Bacillus cereus*. Sm, *Serratia marcescens*.

Among animals wounded with contaminated needles, the global Cox model revealed that the type of food (i.e., whether insects were fed with bacteria, living or dead) had no significant effect on survival, either alone or in interaction with other factors. Ingestion of the living bacteria used here did not result in mortality for *T. molitor*, nor did it induce immune priming. The development stage, bacteria species and the time of septic wounding all significantly influenced the survival of *T. molitor* in a three-way interaction term (**Table 1**; **Figure 1**). Overall, adult survival was lower than larval survival after septic wounding, and the overall mortality rate was higher when individuals were wounded 15 days after the feeding period rather than 24 hours (**Table 1**; **Figure 1**). However, the mortality dynamics induced by septic wounding with the two bacteria species differed according to the insect stage and the timing of the septic wound. Specifically, in larvae, *S. marcescens* was more virulent than *B. cereus* when septic wounding occurred one day after the feeding period, whereas the opposite was observed when the septic wounding occurred 15 days after the feeding treatment (**Figure 1**). No such difference was observed in adults, where *S. marcescens* consistently exhibited higher virulence than *B. cereus* at both post-feeding time points (**Figure 1**).

**Table 1:** Cox model analysis assessing the survival of *T. molitor* larvae and adults that were fed with either inactivated or living bacteria and subsequently subjected to a septic wound with the same bacteria species, either 24 hours or 15 days after the feeding treatment.

Factor	D.F.	L.R. Chi square	P
Stage	1	46.132	**< 0.001**
Bacteria	1	0.446	0.504
Food	2	2.250	0.325
Time	1	11.819	**< 0.001**
Mass within Stage	2	1.558	0.459
Stage*Bacteria	1	0.179	0.672
Stage*Food	2	1.224	0.542
Stage*Time	1	0.754	0.385
Bacteria*Food	2	0.245	0.885
Bacteria*Time	1	24.441	**< 0.001**
Food*Time	2	3.053	0.217
Stage*Bacteria*Time	1	36.534	**< 0.001**

The fixed factors considered included the development stage (‘Stage’: larva or adult), type of food (‘Food’: uncontaminated food, food contaminated with inactivated bacteria, or food contaminated with live bacteria), bacteria species (‘Bacteria’: *Bacillus cereus* or *Serratia marcescens*), and the time of septic wounding (‘Time’: 24 hours or 15 days). Initial body mass (‘Mass’) was nested into the development stage and included as a continuous factor. Additionally, the box in which the insects were maintained was included as a random factor. Values in bold denotes significant results (P < 0.05).

**Figure 2:**
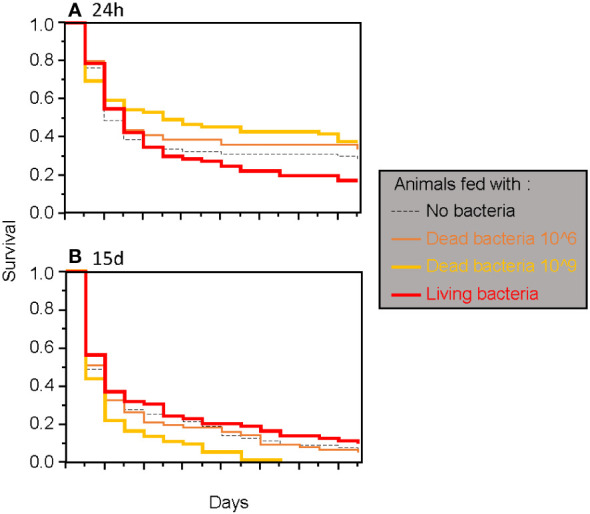
Survival of adult *T. molitor* after a secondary septic wound occurring either 24 hours **(A)** or 15 days **(B)** after the feeding treatment, regardless of the bacteria species used.

**Figure 3:**
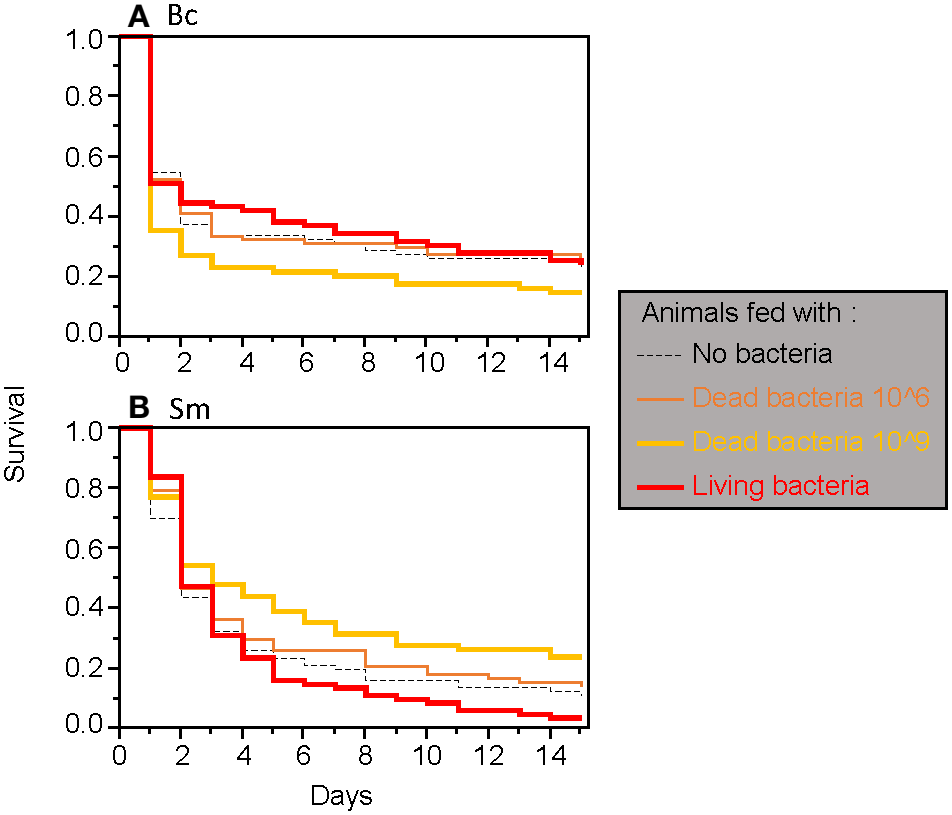
Survival of adult *T. molitor* after a secondary septic wound involving either *Bacillus cereus* **(A)** or *Serratia marcescens* **(B)**, regardless of the timing of the secondary septic wound.

**Figure 4:**
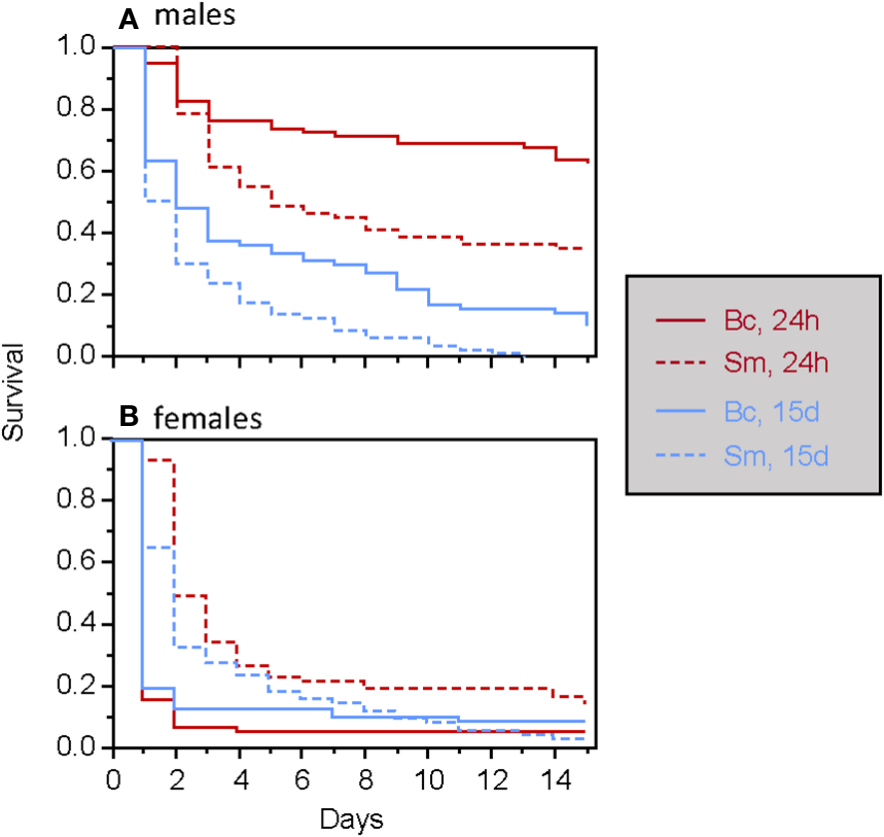
Survival of male **(A)** and female **(B)** *T. molitor* individuals after a secondary septic wound occurring either 24 hours or 15 days, depending on the bacteria species used (Bc, *Bacillus cereus*; Sm, *Serratia marcescens*).

When considering only adults, the type of food was found to have a significant two-way interaction effect with the timing of septic wounding on one hand (**Table 2**; **Figure 2**) and with the bacteria species on the other hand (**Table 2**; **Figure 3**). Specifically, food contaminated with living bacteria had a slight deleterious effect when wounds were made 24 hours after the feeding period but not after 15 days. Meanwhile, the food contaminated with dead bacteria at the highest concentration provided a slight survival benefit when wounds were made after 24 hours but not after 15 days (**Figure 2**). This early protective effect was observed with *S. marcescens* but not with *B. cereus* (**Figure 3**). Sex, bacteria species and the time of wounding all had significant interactive effects (**Table 2**). Females were more sensitive to the infection than males (**Figure 4**), with this difference being more pronounced both when septic wounds were made 24 hours after the feeding period rather than 15 days later (**Figure 4**) and when the wound was inflicted with *B. cereus* rather than with *S. marcescens* (**Figure 4**).

**Table 2:** Cox model analysis assessing the survival of *T. molitor* adults that were fed with either inactivated or living bacteria, and subsequently subjected to a septic wound with the same bacteria species, either 24 hours or 15 days after the feeding treatment.

Factor	D.F.	L.R. Chi square	P
Sex	1	56.451	**< 0.001**
Bacteria	1	6.415	**0.011**
Food	3	0.324	0.956
Time	1	50.038	**< 0.001**
Mass	1	7.362	**0.007**
Sex*Bacteria	1	56.294	**< 0.001**
Sex*Food	3	7.070	0.070
Sex*Time	1	40.869	**< 0.001**
Bacteria*Food	3	19.580	**< 0.001**
Bacteria*Time	1	1.237	0.266
Food*Time	3	12.648	**0.005**
Sex*Bacteria*Time	1	6.169	**0.013**

The fixed factors considered included the individual’s sex (‘Sex’: male or female), type of food (‘Food’: uncontaminated food, contaminated food with inactivated bacteria at 10^6^ cells.mL^-1^, contaminated food with inactivated bacteria at 10^9^ cells.mL^-1^, or contaminated food with living bacteria at 10^9^ cells.mL^-1^), bacteria species (‘Bacteria’: *Bacillus cereus* or *Serratia marcescens*) and the time of septic wounding (‘Time’: 24 hours or 15 days). The initial body mass (‘Mass’) was included as a continuous factor. Additionally, the box in which the insects were maintained was included as a random factor. Values in bold denotes significant results (P < 0.05).

### 3.2 Second experiment: monitoring the ingestion of living bacteria by larvae and its effects

#### 3.2.1 Effect of bacterial content on the amount of ingested food

**Figure 5:**
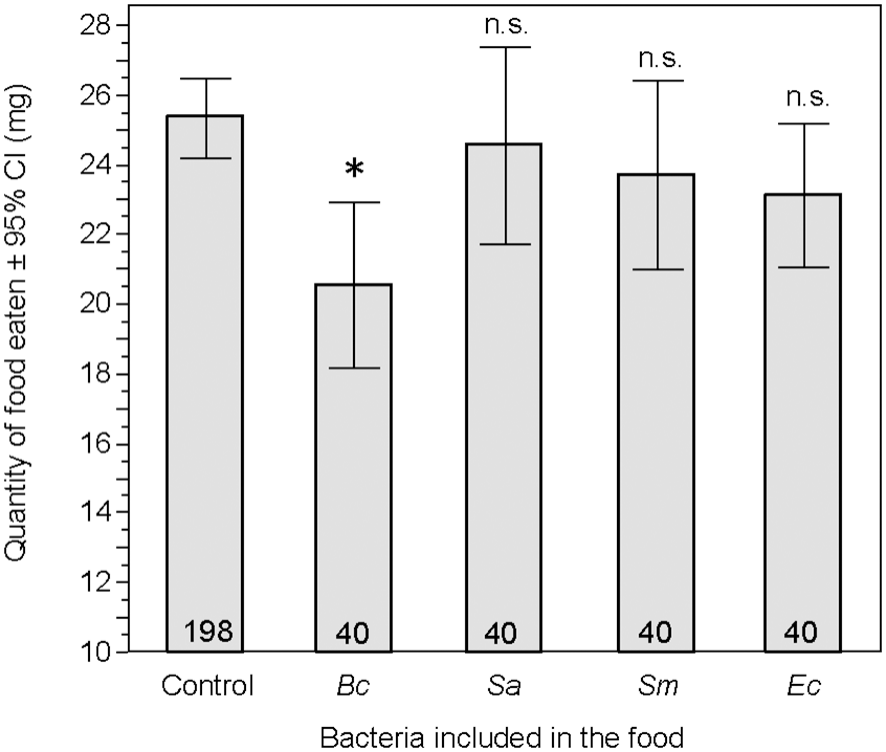
Total quantity of food, in milligrams, eaten by larvae over the three-day feeding period. Treatment groups were compared to the control group. Bc, *Bacillus cereus*; Sa, *Staphylococcus aureus*; Sm, *Serratia marcescens*; Ec, *Escherichia coli*; ns, non-significant difference. *p < 0.05. Numbers in the bars are sample sizes.

At the end of the feeding period, all larvae fed with bacteria consumed a similar amount of feed compared to the control group, except for the group fed with *B. cereus*. These larvae ingested less food than the control group (**Table 3**; **Figure 5**). The initial body mass strongly influenced food intake: the larger the animal, the greater the food intake (**Table 3**). Consistent with this result, we observed that larval mass gain after the feeding period was similar in all groups except for larvae fed with *B. cereus*, which gained less mass (mean ± s.e.: 6.3 ± 6.3 mg) than the control group (9.6 ± 7.6 mg) (**Supplementary Table S4**). Additionally, the initial body mass also significantly influenced mass gain, with the lightest larvae gaining the most mass (**Supplementary Table S4**).

**Table 3:** Linear mixed-effects model analyzing the quantity of food eaten as a function of the food bacterial content (compared to control food without bacteria) and individual body mass (‘Mass’).

Factor	Value	s.e.	t	P
Intercept	13.600	1.684	8.074	**< 0.001**
Food with *B. cereus*	-4.841	1.300	-3.724	**< 0.001**
Food with *S. aureus*	-0.754	1.300	-0.580	0.566
Food with *S. marcescens*	-1.513	1.300	-1.164	0.253
Food with *E. coli*	-1.321	1.306	-1.011	0.320
Mass	0.252	0.034	7.359	**< 0.001**

The box in which the insects were maintained was included as a random factor. Values in bold denotes significant results (P < 0.05).

#### 3.2.2 Effect of food bacterial content on feces quantity and bacteria viability in the gut

**Figure 6:**
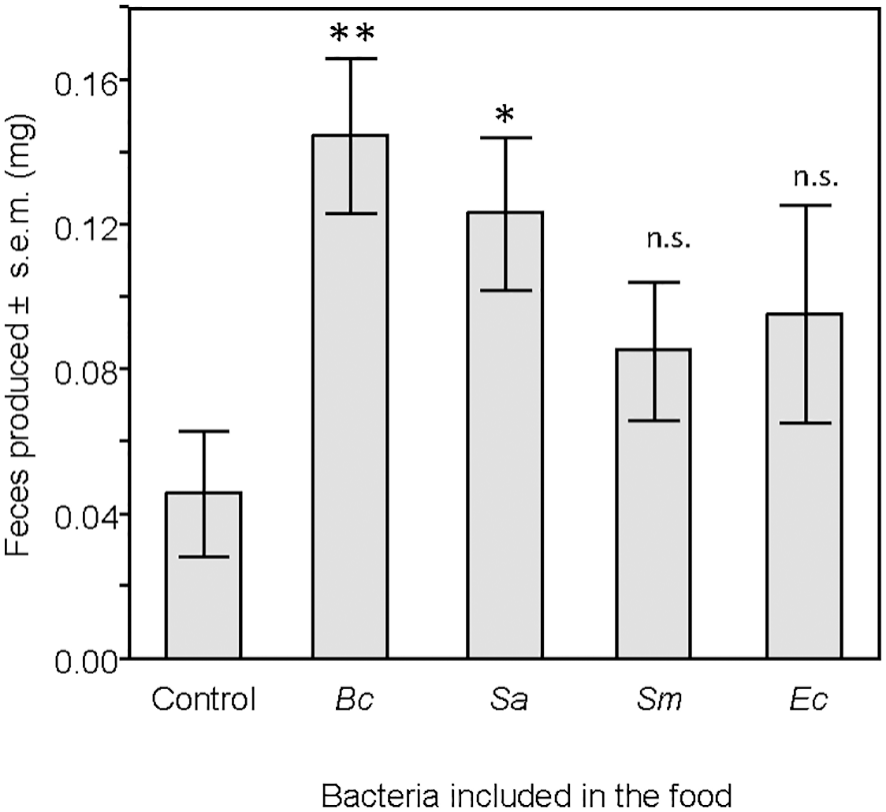
Mass, in milligrams, of feces produced by larvae 24 hours after the end of the feeding period. Treatment groups were compared to the control group. ns, non-significant difference. *p < 0.05. **p < 0.01. N = 20 in each group.

Larvae fed with *B. cereus* and *S. aureus* produced more frass than the control larvae, while no significant difference was found when larvae had eaten food containing *S. marcescens* or *E. coli* (**Table 4**; **Figure 6**).

**Table 4:** Linear model analyzing the quantity of feces produced as a function of the food bacterial content (compared to control food without bacteria) and individual body mass (‘Mass’).

Factor	Value	s.e.	t	P
Intercept	0.103	0.046	2.240	**0.026**
Food with *B. cereus*	0.097	0.031	3.130	**0.002**
Food with *S. aureus*	0.077	0.031	2.527	**0.013**
Food with *S. marcescens*	0.037	0.033	1.146	0.255
Food with *E. coli*	0.049	0.031	1.572	0.119
Mass	-0.001	0.001	-1.416	0.160

Values in bold denotes significant results (P < 0.05).

**Figure 7:**
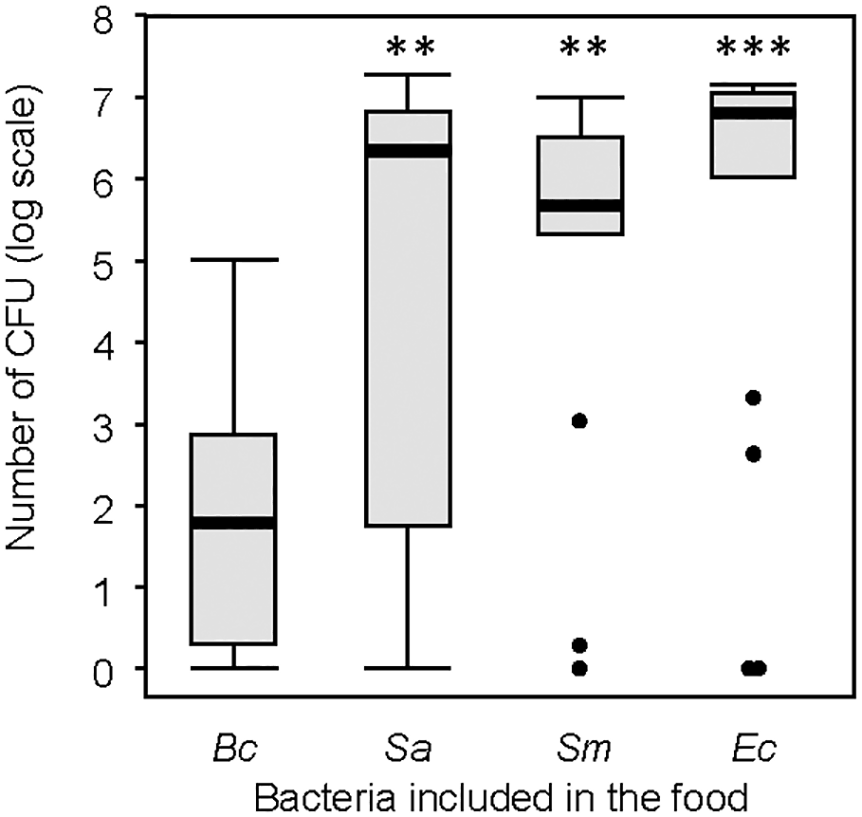
Number of colony-forming units (CFU) from the selected feces samples. Thick lines are the medians, boxes are the upper and lower quartiles, whiskers are the upper and lower interquartile range. Treatment groups were compared to the group including *Bacillus cereus*. ns, non-significative difference. **p < 0.01. *** p < 0.001. N = 20 in each group.

The CFU count revealed the presence of viable bacteria in the feces across all groups (**Figure 7**), confirming their survival during passage through the gut. Among the bacteria, *B. cereus* exhibited the lowest CFU count. Neither the individual body mass of the larvae nor the quantity of feces produced significantly impacted the CFU count (**Supplementary Table S5**).

#### 3.2.3 Effect of food bacterial content on resistance to subsequent bacterial infection

**Figure 8:**
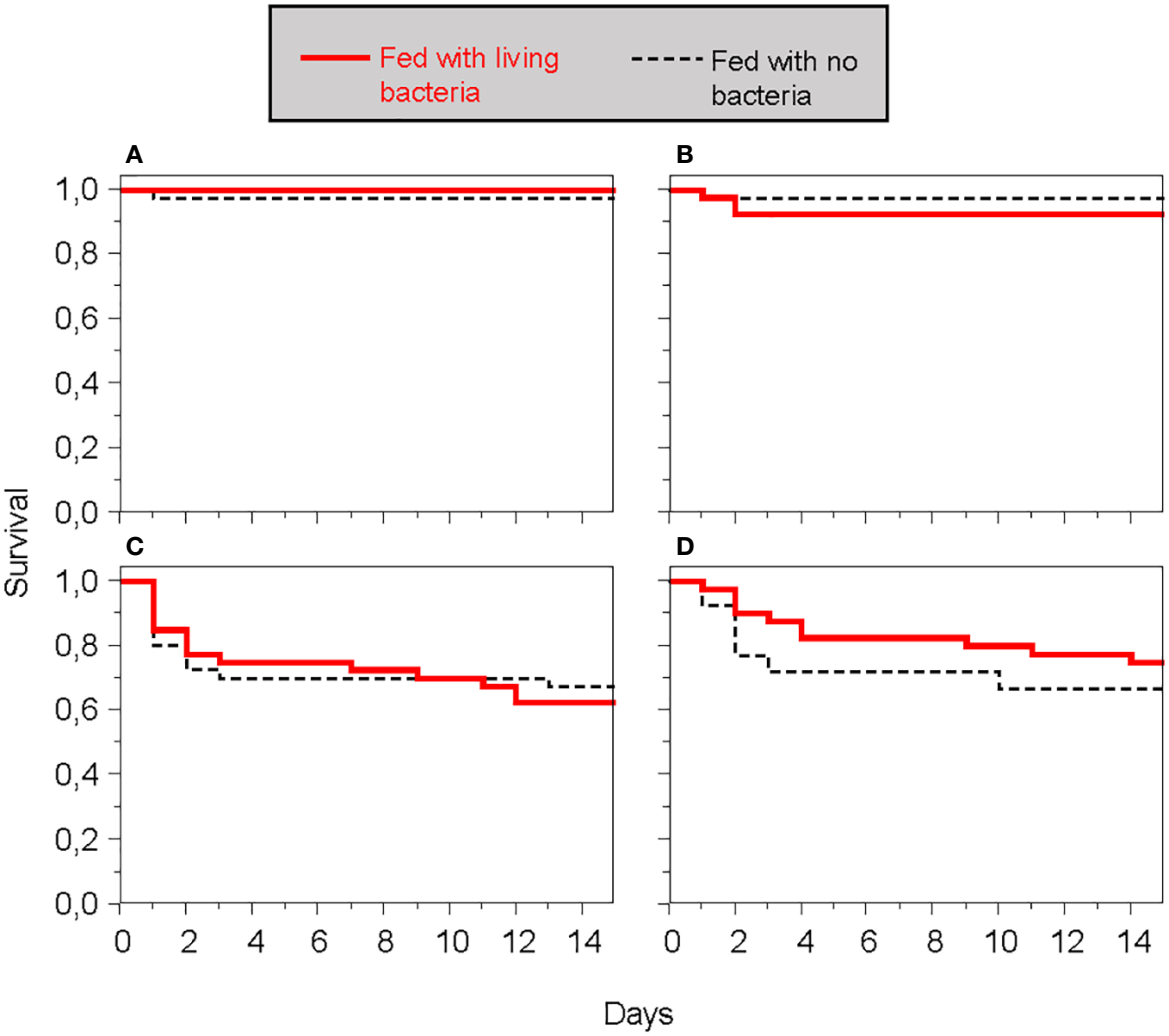
Survival of *T. molitor* larvae to an infection, whether or not individuals were fed with the same bacteria used for infection. **(A)** *Escherichia coli*. **(B)** *Staphylococcus aureus*. **(C)** *Bacillus cereus*. **(D)** *Serratia marcescens*.

Similar to what was observed in the first experiment, larvae fed with bacteria did not exhibit enhanced resistance to subsequent infections with the same bacteria compared to individuals fed without bacteria (**Supplementary Table S6**; **Figure 8**). The various bacteria species induced different mortality rates upon wounding, with *B. cereus* and *S. marcescens* causing higher mortality than *E. coli* and *S. aureus*; the latter two induced almost no death (**Figure 8**).

## 4 Discussion

Our results showed that feeding *T. molitor* larvae and adults with contaminated food did not elicit any long-lasting increased resistance to a second infection involving the same pathogen. However, we noted many differences in insects’ reaction to contaminated food and second infection.

### 4.1 Contaminated food did not kill due to efficient behavioral and gut immunity

In all our experiments, consuming contaminated food with living bacterial pathogens never resulted in a significant increase in mortality among *T. molitor* larvae and adults. We were able confirm the success of the experimental oral infection by retrieving living cells from all the bacterial strains used to contaminate the food in the feces of larvae. This demonstrated that the bacterial pathogens could survive their transit in the gut of the insects and further suggests that the microbes could have expressed their virulence. However, this was not the case. The lack of virulence of the oral infection with *S. aureus* and *E. coli* is rather consistent with the survival outcomes of the septic wounding with these bacterial strains in the present study, as they were unable to significantly kill the larvae through this route of infection, although their pathogenicity was previously found to be not null (15). However, it is surprising that the oral infection with *B. cereus* and *S. marcescens*, proven to be pathogenic for *T. molitor* through septic wounding in this study and in prior ones (30, 31), lacked virulence. The genera *Bacillus* and *Serratia* are indeed well-known for their pathogenicity by both oral and septic injury routes among insects. *S. marcescens* is capable of crossing the gut epithelium in *Drosophila melanogaster* by traversing the enterocytes (32), and some *Bacillus* species are employed in oral infection protocols in insects (11, 21, 33, 34), such as *Bacillus thuringiensis*, which produce crystalline proteins that disrupt the gut epithelium (35–37). In both cases, bacteria can reach the hemocoel, where they multiply and spread throughout the entire insect’s body, leading to death by septicemia. However, *B. cereus* does not produce the aforementioned crystalline proteins (38), which could putatively explain the absence of mortality after the feeding period. Similarly, for *S. marcescens*, since its pathogenicity by the oral route could be strain-specific (32), the strain we used here might not be adapted to induce virulence when ingested by *T. molitor*.

Alternatively, the integument of the gut wall of *T. molitor* could be remarkably efficient in preventing the oral infection by most of the bacterial pathogens, including those used in this study. The gut lining acts as a physical barrier that prevents the entry of pathogens. This barrier could be reinforced by various other mechanisms, such as the patrolling of hemocytes, activation of the prophenoloxidase system and the production of antimicrobial peptides, all of which contribute to the defense against ingested pathogens (39–41). Nevertheless, it is remarkable that a fraction of the bacterial pathogens could survive throughout the gut, suggesting they were eventually able to overcome these defenses.

Interestingly, larvae that consumed food contaminated with *B. cereus* and *S. aureus* produced significantly more feces than control larvae. The insect gut is indeed capable of triggering strong muscle contractions (23) and enterocyte purge (24) against ingested pathogens. Both *B. cereus* (42) and *S. aureus* (43) secrete enterotoxins during their growth, and these might have elicited the observed increase in the defecation rate of the larvae in response to the contaminated food. It is difficult to state whether this induced diarrhea benefits the pathogens or the host. On the one hand, increased frass production might benefit the bacterial pathogen in spreading in the environment after being ingested by the host and thus increasing the transmission rate. The observation of living bacterial cells in the frass could be consistent with this hypothesis. However, the fact that the amount of frass released is not positively correlated with the number of CFUs observed may not support this hypothesis. On the other hand, an increased defecation rate might benefit the contaminated larvae in that it allows them to rapidly get rid of pathogens before they can cross the gut wall. Increased defecation after pathogen ingestion, therefore, seems to be a physiological defense mechanism, albeit less specific than the reluctance behavior we also observed.

Indeed, we observed that larvae exposed to food contaminated with *B. cereus* were those that consumed the least amount of food compared to the other larval groups. This resulted in a lower mass gain than in other larvae. *B. cereus* is a Gram(+) entomopathogen bacterium proven to be one of the deadliest bacterium we used in this experiment through septic wounding. By contrast, food contaminated with *S. marcescens*, another deadly entomopathogen but Gram(-) bacterium, was consumed as much as control food, as were foods contaminated with *S. aureus* and *E. coli*, generalist bacteria inducing almost no virulence in our assays. Previous studies suggested that Gram(+) bacterial pathogens, particularly Bacilli, might have played a specific role in shaping immunity of the mealworm beetle with regard to the evolution of immune priming (15, 44–46). Our results confirm that these bacteria are recognized as an important threat for the mealworm beetle, which may also have evolved the ability to specifically recognize contaminated food and adjust its feeding behavior accordingly. Such results are also in line with the work of Guo et al. (26) who tested *T. molitor* larvae preference for food contaminated with different fungal pathogens, and found that larvae reduced their feeding rate when pathogens inducing the greatest mortality rates were present in their food.

Therefore, we provided evidence that genuine oral infections did not lead to death of the mealworm beetle, even when using high concentrations of authentic entomopathogenic bacterial strains known to be virulent upon wounding. Behavioral and gut immunity responses appear to greatly contribute to their survival to oral infection, although further investigation is necessary. These findings imply that these latter defense mechanisms are probably effective in mitigating the selective pressure that these pathogens could have exerted during oral infection, suggesting that the development or activation of other defensive traits may not be necessary.

### 4.2 Oral bacterial contamination did not induce long-term immune priming

Neither *B. cereus* nor *S. marcescens* induced any long-lasting protective effect in larvae or adults that ingested living or dead bacteria prior septic wounding. This sharply contrasts with the long-term immune priming conferred by dead bacterial injection (15, 46). Immune priming is therefore probably effective only if pathogens or antigens have reached the hemocoel of insects, where they are in direct contact with circulating hemocytes, important components of immune activation (14). Gut immunity (see above) has likely limited, if not prevented, the passage of the bacterial pathogens across the gut wall into the hemocoel.

We observed a slight beneficial effect of contaminated food only in adults exposed to septic wounding one day after the feeding period. This short-time effect was observed only with food containing high dose of inactivated bacteria (10^9^ cells.mL^-1^) and only with *S. marcescens*. This result resembles that of González-Acosta et al. (22) at first sight but differed in many ways. It was not observed in larvae in our case and occurred only when bacteria were provided at a high concentration, while the aforementioned authors found the same result at a lower concentration (22). The observed immune activation in adults might be explained by variations in the allocation of resources to basal immunity based on the developmental stage, in accordance with some of the findings discussed below: given that larvae typically exhibit higher constitutive immunity on average compared to adults (see **Figure 1**), their capacity to rapidly boost their immune activity after bacterial ingestion may be limited. In addition, in the long term, the survival pattern exhibited a reversal: if a septic wound occurred later, the adults with lower survival rates were those that had ingested high doses of dead bacteria. Therefore, the ingestion of high doses of dead bacteria may induce a short-term increase in the immune response, but this enhancement appears to come at a long-term cost. This slight elevation in the short-term immune response was observed only after the ingestion of high doses of *S. marcescens*, whereas similar high doses of dead *B. cereus* were slightly deleterious. Therefore, the stimulation of the adult’s immune system appears to be pathogen-dependent, as is the long-term immune priming after injected vaccination (15, 46).

The survival of *T. molitor* after septic wounding is complicated by our evidence of stage-dependent sensitivity to the wounding itself. While larvae did not experience increased death from sterile wounding (wounding without our experimental bacteria), adults pricked with a sterile needle 15 days after the feeding period exhibited a decreased survival compared to those pricked one day after the feeding period. The decrease in resistance associated with aging might account for this result, as suggested in the study of Daukšte et al. (47). In their experiment, the implantation of sterile nylon filaments in *T. molitor* adults which were 11 days or 61 days old resulted in higher mortality in the latter group compared to the former. However, the pattern of immunosenescence is variable among components of the immune system. For example, the decline with age is more pronounced for cellular defenses than for antibacterial defenses, and the activity of the prophenoloxidase system is even higher in older animals than in younger ones (48). The activation of the prophenoloxidase is known to induce immunopathology in insects (49), which could explain the higher mortality in older insects after wounding or defense against inactive foreign bodies due to age-related immunopathology (50, 51). It remains to be tested if the fourteen-day gap between our youngest and older animals is sufficient to explain such a difference, but this result should be considered when interpreting the effect of septic wounding in adults described below. Nonetheless, the progression of mortality varied between septic and sterile wounding in adults: mortality occurred rapidly and sharply in septic conditions, whereas it was more gradual in sterile conditions (**Figure 1**, **Supplementary Figure S1**). Therefore, microbes caused death more rapidly than wounding itself.

Hence, despite the complexity of our results, particularly in adults, they provide reasonable evidence that immune priming in *T. molitor* could not be induced through the oral infection. Moreover, the observation that contaminated food in larvae could trigger a gut immunity response implies that the detection of pathogens in the gut was acknowledged, and there was potential for the stimulation of other defense mechanisms, such as immune priming. However, this was not the case. These results, together with previous findings demonstrating that immune priming could be induced through injection or septic wounding (4, 15, 46, 52), clearly suggest that the oral route of contamination by bacterial pathogens represents a marginal selective pressure for the evolution of immune priming in *T. molitor*. In contrast, septic wounding appears to have played a more significant role. In a way, we could propose that microbe ingestion does not represent a “danger” *sensu* Matzinger (53), whereas septic wounding could stimulate a higher immune response (54).

### 4.3 Comparison of mortality and immune priming effect between development stages and among adults

Our first experiment revealed that adults were more sensitive to septic wounding than larvae. In many insect species, immature stages are expected to be more resistant than adult stages. Indeed, strong selection pressures for high constitutive immunity should be at work in young individuals since it favors the probability that they reach adulthood and reproduce, while adults must balance their energy expenditure between immune activity and reproduction, thus leading to a decrease in immune system efficiency after the imaginal emergence (55). We also observed a switch of virulence of *B. cereus* and *S. marcescens* between the two time points among the larvae. This result remains puzzling and unexplained since we never observed such a variation in differences in pathogenicity between these two bacteria species in any of our preliminary experiments. Perhaps a variation between the bacterial growths between the two time-points can explain such a difference.

When comparing mortality among adults, we found that females were a more sensitive to infections than males. This result is somewhat unexpected given that, based on life history theories, females are expected to invest more in immune defenses than males. This investment is thought to optimize their life-span and reproduction (56–59). This theory has already been validated in various invertebrate species (60–64). However, Dhinaut et al. (15) also discovered that *T. molitor* males were more resistant than females to infections. Additionally, it was observed that males exhibited higher phenoloxidase activity than females (65). One could argue that, given *T. molitor* females’ ability to reproduce over a relatively long period considering their lifespan (31), reproduction may alter the immune system efficiency. Data showed that reproduction throughout the entire lifespan does indeed reduce immune system activity, but this response is not consistent for all components of the immune system (31). In addition, we found here that males are more resistant than females to *B. cereus*, and that *S. marcescens* is globally deadlier than *B. cereus*. Dhinaut et al. (15) found a similar result when comparing the sensitivity of *T. molitor* adults to *B. thuringiensis* and *Serratia entomophila*, two entomopathogens very closely related to the ones we used. Not only does it further confirm that males are more resistant to females in *T. molitor*, but it is also in line with the hypothesis proposed by Dhinaut et al. (15) and Dubuffet et al. (44) that this insect species evolved better resistance to Gram(+) bacteria than Gram(-) bacteria through immune priming.

## 5 Conclusion

Our study indicates that immune priming is not induced by the consumption of bacteria in *T. molitor* larvae and adults, in contrast to studies involving pathogen injection. We therefore propose that immune priming may not have evolved through the oral contamination route in *T. molitor*. To validate this hypothesis, further investigations using other pathogen species in feeding assays are necessary. In addition, we observed that larvae exhibited a reluctance behavior and a physiological reaction (i.e., increased defecation) in response to the Gram(+) entomopathogen *B. cereus*. These findings provide new insights into *T. molitor*’s ability to mitigate infection risks imposed by highly threatening pathogens, potentially alleviating selective pressure on immune priming following oral infections.

## Data availability statement

The raw data supporting the conclusions of this article will be made available by the authors, without undue reservation.

## Ethics statement

The manuscript presents research on animals that do not require ethical approval for their study.

## Author contributions

AG: Conceptualization, Data curation, Formal analysis, Investigation, Methodology, Validation, Visualization, Writing – original draft, Writing – review & editing. CD: Investigation, Methodology, Project administration, Writing – review & editing. AB: Investigation, Methodology, Writing – review & editing. TR: Conceptualization, Data curation, Formal analysis, Funding acquisition, Investigation, Methodology, Project administration, Resources, Supervision, Validation, Visualization, Writing – original draft, Writing – review & editing. YM: Conceptualization, Data curation, Formal analysis, Funding acquisition, Investigation, Methodology, Project administration, Resources, Supervision, Validation, Visualization, Writing – original draft, Writing – review & editing.
