# Supplementary material for: Infection risk by oral contamination does not induce immune priming in the mealworm beetle (Tenebrio molitor) but triggers behavioral and physiological responses
Source: Front Immunol. 2024 Feb 8;15:1354046. doi: 10.3389/fimmu.2024.1354046 (PMC10885348; doi:10.3389/fimmu.2024.1354046)
Supplement: Supplementary file 1 [file DataSheet_1.docx]

**Table S1:** Sample size of the first feeding experiment, where *T. molitor* at larval or adult stage fed with different types of food were wounded either 24 hours or 15 days after the feeding treatment, with a needle either sterile or contaminated with living bacteria of the same species used for feeding. This experimental design was made with two bacteria species: *Bacillus cereus* and *Serratia marcescens*.

|  | **Control food** | **Food with living bacteria** | **Food with inactivated bacteria,**  **10^6^cells.mL^-1^** | **Food with inactivated bacteria,**  **10^9^cells.mL^-1^** |
| --- | --- | --- | --- | --- |
| **LARVAE** |  |  |  |  |
| **Not wounded** | 20 |  |  |  |
| **Wounded 24h post-feeding** |  |  |  |  |
| Wound with bacteria | 40 | 40 | 40 |  |
| Sterile wound | 20 |  |  |  |
| **Wounded 15 days post-feeding** |  |  |  |  |
| Wound with bacteria | 40 | 40 | 40 |  |
| Sterile wound | 20 |  |  |  |
| **ADULTS*** |  |  |  |  |
| **Not wounded** | 20 |  |  |  |
| **Wounded 24h post-feeding** |  |  |  |  |
| Wound with bacteria | 40 | 40 | 40 | 40 |
| Sterile wound | 20 |  |  |  |
| **Wounded 15 days post-feeding** |  |  |  |  |
| Wound with bacteria | 40 | 40 | 40 | 40 |
| Sterile wound | 20 |  |  |  |

* Each group includes ½ males ½ females

**Table S2:** Sample size of the second feeding experiment, where *T. molitor* larvae fed with different types of food were wounded 15 days after the feeding treatment, with a needle either sterile or contaminated with living bacteria of the same species used for feeding. This experimental design was made with four bacteria species: *Bacillus cereus*, *Staphylococcus aureus*, *Serratia marcescens* and *Escherichia coli.*

|  | **Control food** | **Food with living bacteria** |
| --- | --- | --- |
| **LARVAE** |  |  |
| **Not wounded** | 20 |  |
| **Wounded 15 days post-feeding** |  |  |
| Wound with bacteria | 40 | 40 |
| Sterile wound | 20 |  |

**Table S3:** Wald test established from a Cox model analyzing the survival of *T. molitor* adults fed with carrot without bacteria, and then wounded with a sterile needle (compared to non-wounded adults). The age of wounding (24 hours / 15 days) is a fixed factor. The initial mass was included as a continuous factor, and the box of rearing was included as a random factor.

| Factor | coef | exp(coef) | se(coef) | z | P |
| --- | --- | --- | --- | --- | --- |
| Wounded at 24 h | 1.437 | 4.207 | 1.119 | 1.28 | 0.200 |
| Wounded at 15 days | 3.553 | 34.912 | 1.044 | 3.40 | **< 0.001** |
| Initial mass | -0.021 | 0.979 | 0.014 | -1.47 | 0.140 |

**Table S4:** Linear mixed-effects model analyzing the effect of food bacterial content (compared to control food without bacteria) and individual mass on the normalized mass gain. The box of rearing was considered as a random factor.

| Factor | Value | s.e. | D.F. | t | P |
| --- | --- | --- | --- | --- | --- |
| Intercept | 0.362 | 0.036 | 323 | 10.192 | **< 0.001** |
| Food with *B. cereus* | -0.074 | 0.027 | 31 | -2.702 | **0.011** |
| Food with *S. aureus* | 0.007 | 0.027 | 31 | 0.261 | 0.796 |
| Food with *S. marcescens* | -0.003 | 0.027 | 31 | -0.116 | 0.908 |
| Food with *E. coli* | -0.002 | 0.028 | 31 | -0.057 | 0.955 |
| Initial mass | -0.003 | 0.001 | 323 | -4.380 | **< 0.001** |

**Table S5:** Generalized linear model with quasi-Poisson distribution analyzing the effect of food bacterial content (compared to food containing *Bacillus cereus*), quantity of food ingested, feces mass and individual initial mass on the quantity of bacterial colony forming units (CFU).

| Factor | Estimate | s.e. | t | P |
| --- | --- | --- | --- | --- |
| Intercept | 2.023 | 0.894 | 2.263 | **0.027** |
| Food with *S. aureus* | 2.772 | 0.834 | 3.322 | **0.001** |
| Food with *S. marcescens* | 2.676 | 0.836 | 3.202 | **0.002** |
| Food with *E. coli* | 3.425 | 0.819 | 4.180 | **<0.001** |
| Food ingested | 0.073 | 0.016 | 4.698 | **<0.001** |
| Initial mass | -0.014 | 0.010 | -1.395 | 0.167 |
| Feces mass | 1.065 | 0.719 | 1.481 | 0.143 |

**Table S6:** Cox model analyzing the survival of *T. molitor* larvae fed with living bacteria, and then wounded with the same bacteria species. Fixed factors are the type of food (no bacteria in the food / living bacteria added to the food), bacteria species and their interaction. The initial mass was included as a continuous factor, and the box of rearing was included as a random factor.

| Factor | D.F. | L.R. Chi square | P |
| --- | --- | --- | --- |
| Food type | 1 | 0.022 | 0.882 |
| Bacteria species | 3 | 41.604 | **<0.001** |
| Food*Species | 3 | 1.044 | 0.791 |
| Initial mass | 1 | 5.684 | **0.017** |


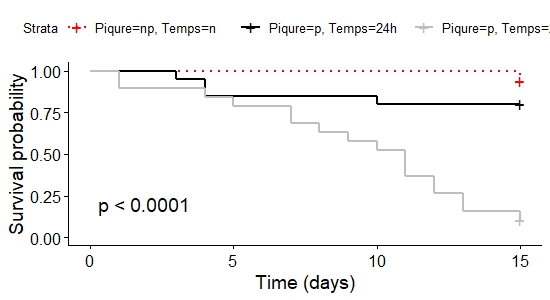


**Fig. S1:** Survival of *T. molitor* adults fed with carrot without bacteria, not wounded (dotted red line), or wounded with a sterile needle 24 hours (black line) or two weeks (grey line) after the feeding period.
